# Manual Control Age and Sex Differences in 4 to 11 Year Old Children

**DOI:** 10.1371/journal.pone.0088692

**Published:** 2014-02-11

**Authors:** Ian Flatters, Liam J. B. Hill, Justin H. G. Williams, Sally E. Barber, Mark Mon-Williams

**Affiliations:** 1 Institute of Psychological Sciences, University of Leeds, Leeds, West Yorkshire, United Kingdom; 2 Department of Mental Health, University of Aberdeen, Royal Cornhill Hospital, Aberdeen, Scotland, United Kingdom; 3 Bradford Institute for Health Research, Bradford Teaching Hospitals NHS Foundation Trust, Bradford, West Yorkshire, United Kingdom; University of Reading, United Kingdom

## Abstract

To what degree does being male or female influence the development of manual skills in pre-pubescent children? This question is important because of the emphasis placed on developing important new manual skills during this period of a child's education (e.g. writing, drawing, using computers). We investigated age and sex-differences in the ability of 422 children to control a handheld stylus. A task battery deployed using tablet PC technology presented interactive visual targets on a computer screen whilst simultaneously recording participant's objective kinematic responses, via their interactions with the on-screen stimuli using the handheld stylus. The battery required children use the stylus to: (i) make a series of aiming movements, (ii) trace a series of abstract shapes and (iii) track a moving object. The tasks were not familiar to the children, allowing measurement of a general ability that might be meaningfully labelled ‘manual control’, whilst minimising culturally determined differences in experience (as much as possible). A reliable interaction between sex and age was found on the aiming task, with girls' movement times being faster than boys in younger age groups (e.g. 4–5 years) but with this pattern reversing in older children (10–11 years). The improved performance in older boys on the aiming task is consistent with prior evidence of a male advantage for gross-motor aiming tasks, which begins to emerge during adolescence. A small but reliable sex difference was found in tracing skill, with girls showing a slightly higher level of performance than boys irrespective of age. There were no reliable sex differences between boys and girls on the tracking task. Overall, the findings suggest that prepubescent girls are more likely to have superior manual control abilities for performing novel tasks. However, these small population differences do not suggest that the sexes require different educational support whilst developing their manual skills.

## Introduction

Large population-based studies of children reliably find sex differences for specific cognitive functions [1], [2]. Girls outperform boys on standardised tests of attention, emotion recognition, verbal and facial memory tasks. Boys outperform girls on sensorimotor, visuo-spatial and mathematical problem-solving tasks. These findings complement neuroimaging research showing structural differences in the developmental trajectories of the male and female brain [3] and a clinical literature which indicates an increased prevalence of certain neurodevelopmental disorders in males [4], [5]. Nonetheless, evidence from meta-analyses [6] suggests that the importance of such sex differences is often overstated. Hyde et al. [6] argued in favour of a ‘gender similarities hypothesis,’ pointing out that the sexes are similar in many more facets of their psychological functioning than they are dissimilar. Sex differences in cognition are often task-specific, small in magnitude and/or show high inter-individual variability [7], leading Hyde et al. to suggest that they are of limited value as heuristics for explaining individual children's everyday behaviours. Moreover, ‘media sensationalising’ of relatively innocuous sex differences is argued to have profoundly negative consequences [8]. For example, male advantages on visuo-spatial tasks are repeatedly used as a reductive excuse for the under-representation of females in mathematical and scientific professions [7], [9]. Consequently, it is important we gain a clearer understanding of the degree to which sex genuinely affects children's development because this will allow us to adopt appropriate teaching strategies (e.g. recognising significant differences or encouraging inequality within specific curriculum areas, which may vary with age).

In particular, there is currently insufficient clarity over the role that sex may play in the development of children's manual motor skills. This is despite the topic being of fundamental educational importance. For example, children's handwriting development is heavily influenced, amongst other factors, by their underlying manual dexterity [10]. Measures at school entry of children's fine-motor skills (i.e. activities distinguished through their requirement for a high-degree of precision and typically involving some form of manual object manipulation [11]) are predictive of children's later academic performance [12], [13]. Meanwhile, for children diagnosed as having coordination difficulties, some of the most frequently experienced functional impairments are for academically relevant manual tasks such as producing written work, drawing and in-hand manipulation of materials [14]–[16].

There is some evidence for sex differences in motor-skill development if ‘motor skill’ is treated as a homogenous ability. Epidemiological studies have found that development coordination disorder (DCD) is at least twice as common in boys than girls [5], [17], [18]. If DCD is simply a characterisation of the motor skills of children at one end of a continuum, then sex differences in a clinical population might reflect differences in development within the typical population. In contradiction to this notion, Malina, Bouchard and Barr-Or [11] report that sex differences in the rate of acquisition of recognised motor-milestones during infancy are few, inconsistent and possibly culturally determined. Once adolescence is reached sex-differences in gross-motor skills (i.e. activities involving locomotion and movement of the torso [11]) are relatively well established. For example, there is good evidence of males performing better on large-object control tasks, in particular on tests of throwing and striking ability [11], [19]–[23], with these performance gaps widening with age [24]. However, such emerging male advantages are thought to be primarily determined by post-pubescent anatomical sex differences (e.g. relatively greater increases in muscle tissue in males) and cultural biases towards males being more likely to engage in activities that selectively develop their object-control skills (e.g. ball sports) [22], [24]. Thus, collectively these findings do little to enlighten our understanding of how sex may influence fine-motor manual control development, particularly in the period between infancy and pubescence, in typically developing children.

Fine-motor skills can be considered a specialised sub-category of motor behaviour [25], which include manual tasks that are less dependent on muscular strength but are more directly relevant to academia. In relation to such tasks, Gur et al. [2] reported that males were faster in basic speeded manual responses in a sample of 3,500 youths from 8 to 21 years old (tasks involved repeated finger tapping, moving a computer mouse to click on a square that appeared at unpredictable on-screen locations). These advantages did not emerge until adolescence though and non-speed related outcomes (e.g. accuracy) were not analysed. Gur et al.'s results agree with a smaller cross-sectional study (n = 106, 9 to 17 year olds) that found a male advantage for learning manual sequences (finger-tapping sequences) [26]. However, in contrast, Poole et al. [27] reported that girls were quicker in a task which required participants to insert and remove pegs from a wooden board as quickly as possible, using their preferred then non-preferred hand (n = 406 from 4-19 year olds). Two studies [20], [28] have reported that between the ages of 7 and 12 years girls outperformed boys on a standardised pencil-and-paper battery of manual dexterity tasks (from the Movement ABC-2 assessment battery [29]). Sex differences were also observed on pencil-and-paper handwriting tasks examined during one of these studies [28] - a female advantage for quality but not speed of writing being found in a sample (n = 131) of 7–12 year olds. Once more, these results conflict with a comparable study (n = 127) that reported no sex-differences in 5–12 year-olds on a similar pencil-and-paper drawing task [30]. In sum, no consistent picture emerges from the findings of the previous studies that have investigated sex differences in children's manual control abilities. In part, this may be due to various methodological limitations: the computerised methodologies discussed only assess manual skill with respect to speed but not movement quality [2], [27], whilst alternative pencil-and-paper based assessments [20], [28] have been criticised for relying on subjective scoring methods to evaluate movement quality [31].

A technologically innovative approach to overcoming these limitations is to use digital tablets to record manual movements (see [26], [31]–[35]). This methodology typically involves participants using a stylus to interact with a tablet PC (like using a pen with paper) and has the advantages of being able to assess both the speed and quality of participant's kinematic responses, objectively and in detail. This approach has particular ecological validity for investigating those aspects of manual control that are important for in-hand stylus manipulation (an aspect of manual dexterity that contributes to ones' handwriting and drawing abilities). Studies using tablet technology (not always to explicitly address the issue of sexual dimorphism) report mixed results regarding sex differences. Dorfberger et al [26] reported that girls were significantly faster at writing nonsense words in early blocks of trials (n = 116, 9–17 years age range) but this effect disappeared in later blocks before a male advantage appeared in the final blocks for the oldest age group only (17 years). Rueckreigel et al [32] reported that males were faster in a drawing task (producing a circle) but not on a sentence or repetitive letter writing task (n = 187, 6–18 years old), though the study did not stratify the sample for age. Van Mier [33] found no sex differences in a task that required children to move a handheld stylus around small and large targets on a screen (n = 60, 4–12 years age range). Blank et al [34] also found no sex differences on a task requiring the repetitive drawing of straight lines and circles (n = 53, 7–14 years age range). Genna & Accardo [35] found a small female advantage in younger age-groups when carrying out five cursive handwriting tasks (n = 208, 7–14 years age range). There are difficulties with interpreting these results though because the age ranges frequently included pre- and post-pubescent children and some of these tasks are also arguable confounded by having a degree of familiarity and cultural dependence (i.e. require prior knowledge of letters, words, grammar).

It is clear that the issue of pre-pubescent sex differences in fine motor manual control has yet to be directly investigated. In order to address this issue, we measured performance in children aged 4–11 years as this age range can be considered pre-pubescent with reasonable confidence. Moreover, this age range corresponds to ‘primary schools’ within the UK educational system – schools where the focus is on developing core manual skills such as handwriting and drawing. To allow us to examine manual control objectively, with respect to both its speed and quality, in a large community based sample, we employed a portable digital tablet system capable of providing detailed kinematic information on how children interacted, using a stylus, with visual stimuli presented on a tablet PC's screen.

In deciding upon the particular battery of tasks we presented to participants via the tablet system, we were mindful of the variety of assessment methods used in the previous research. A very broad range of tasks can be used to assess fine-motor control (e.g. manual response reaction time tasks, manual sequence learning, writing and drawing tasks), with performance on any of them dependent in large part on prior experience in the specific task. Nevertheless, a common feature of many of these canonical ‘fine-motor’ tasks is that they require precise ‘hand-eye coordination’. Such visuo-manual control is often discussed as being particularly important in manual tasks requiring object manipulation [36]–[39]. Combining this consideration with the fact tablet methodology lends itself to presenting tasks that involve in-hand manipulation of a stylus; we focussed our investigation on testing basic visuo-manual control skills that are likely to underpin a child's proficiency for controlling a stylus. We created three novel tasks that involved controlling a handheld stylus. These tasks, whilst being novel and therefore hopefully as free as possible from cultural bias, encompassed many of the functional challenges present in everyday tasks requiring stylus use, namely: tracking moving targets, tracing shapes and making aiming movements. These tasks tap into specific control mechanisms (tracking relies on the ability to predict target movement, tracing shapes requires precise force control whilst aiming movements rely on accurate feed forward mechanisms and fast implementation of online corrections).

We reasoned that testing a large number of children on this task battery would allow us to draw some solid conclusions regarding the degree to which sex influences the development of ‘manual control’ abilities within pre-pubescent children, in particular those relevant to learning to manipulate a stylus manually. On the basis of the ‘gender similarities hypothesis’ [6], we predicted we might find small but significant differences in manual control between the sexes. Furthermore, given evidence of gross-motor sex differences increasing with age during adolescence [24], we considered it probable we might find age related improvement in manual-control, during pre-pubescence – improvements that were moderated by sex.

## Methods

### Participants

Participants were recruited from two primary schools in West Yorkshire, UK. A total of 422 out of 484 students agreed to participate (the others were either absent on the day of testing or did not give personal or parental consent). Table 1 provides descriptive statistics for the age, sex, handedness and distribution across categorical age-bands. Informed written consent was obtained from Head-teachers of the participating schools (acting in loco parentis for their students). Additionally, each school also internally obtained informed consent from the individual parents/guardians of the children, giving them advanced notice of the study and their right to opt out should they wish to do so. After having the study explained to them, children gave their verbal consent immediately prior to participating (written consent being impractical for all children within this age-range). The University of Leeds Ethics and Research committee approved these consent procedures and all other aspects of the study's design and methodology. The study was performed in accordance with the ethical standards laid down in the 1964 Declaration of Helsinki.

**Table 1:** Descriptive statistics for age, sex and handedness of whole sample and across age-bands.

	Total Sample		4 to 5 years		6 to 7 years		8 to 9 years		10 to 11 years	
**n**	422		80		122		143		77	
**Sex** 1										
Male	216	(51%)	40	(50%)	60	(49%)	80	(56%)	36	(47%)
Female	206	(49%)	40	(50%)	62	(51%)	63	(44%)	41	(53%)
**Handedness** 1										
Right	369	(87%)	71	(89%)	111	(91%)	123	(86%)	64	(83%)
Left	53	(13%)	9	(11%)	11	(9%)	20	(14%)	13	(17%)
**Age (years, months)**										
Median	8,1		5,4		7,2		9,1		10,7	
IQR	6,6 to 9,8		4,10 to 5,9		6,7 to 7,6		8,7 to 9,7		10,4 to 11,0	
Range	4,6 to 11,5		4,6 to 5,11		6,0 to 8,0		8,0 to 10,0		10,0 to 11,5	

1 Denominators for percentages are relative to each column's n (see first row of the table).

### Materials

The test battery was designed and presented using the Clinical Kinematic Assessment Tool (CKAT), a custom software package specialised for presenting interactive visual stimuli on a tablet laptop computer screen, whilst simultaneously recording participant's kinematic responses to these stimuli via interactions with the screen using a handheld stylus (see Culmer, Levesley, Mon-Williams and Williams [31] for a description of the underlying architecture). CKAT was implemented on Toshiba tablet portable computers (Portege M700-13P, screen size: 303×190 mm, 1280×800 pixels, 32 bit colour, 60 Hz refresh rate) with a pen-shaped stylus (140×9 mm diameter) used as an input device. For every trial within every subtest the position of the stylus was recorded at a rate of 120 Hz, with a 10 Hz dual-pass Butterworth filter applied to the raw positional data at the end of each testing session.

The CKAT software then calculated a range of appropriate spatial, temporal and frequency-based kinematic metrics that described a participant's movements in detail during each trial. Spatial indices, such as total distance moved by the stylus and relative difference between actual distance moved and an ideal trajectory, were calculable to provide information about the accuracy and efficiency of participants' movements. Temporal indices for point-to-point component movements gave information on the duration between participants starting and ending specific movements and their velocity profile. Frequency indices were used to provide information on dynamic ‘tracking-type’ movements, indicating how closely a participant could mimic a target frequency whilst tracking a target moving in a sinusoidal motion (see Culmer et al. for more details [31]).

### Procedure

Participants were seated at a table of appropriate height for their age. A tablet computer in landscape orientation was placed in front of them with its screen folded flat. The edge of the tablet nearest the participant was 15 cm from the table's edge. Participants were instructed to hold the stylus in their dominant hand and were explicitly asked not to switch the stylus between hands during testing or use both hands to bimanually manipulate the stylus. They were instructed to, as much as possible, keep their non-dominant hand stationary, on the table top, off to the side of where the tablet had been placed.

The ‘testing stations’ were placed around the periphery of a large classroom, with one researcher sat to the side of each station. This arrangement allowed for groups of participants to be tested simultaneously. To minimise distractions during testing, stations were separated by at least 2 metres, participants faced away from one another and direct sources of light were removed to minimise reflection on the tablet screen. For each participant, the battery was completed in a single session lasting approximately 12–15 minutes. The test battery comprised of three sub-tests, presented to all participants in the following fixed order:

#### Tracking (without and with a spatial guide)

**Figure 1:**
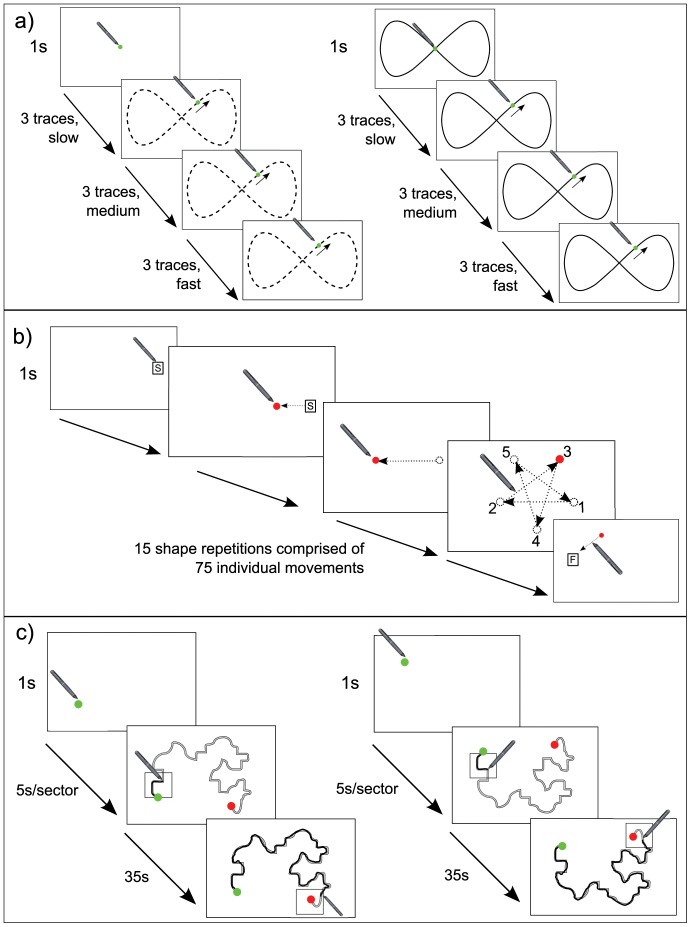
Illustrations of the three manual control battery tasks: (a) Tracking, (b) Aiming and (c) Tracing. (a) Left is a schematic of first Tracking trial (i.e. without ‘Guideline’), annotated with a dotted line to indicate the trajectory of the moving dot. Right is a schematic of the second Tracking trial, which included the additional Guideline. (b) Schematic of the Aiming subtest, annotated with dotted arrows implying the movements participants would make with their stylus to move off the start position, between target locations and to reach the finish position. On the 4^th^ panel further annotations indicate the locations in which targets sequentially appeared, with numbers indicating the sequence in which they were cued. (c) Left is a schematic depicting tracing path A and right is a schematic depicting tracing path B. The black shaky lines are an example of the ‘ink trails’ a participant would produce with their stylus in the course of tracing.

This sub-test comprised of two trials. In the first, participants began by placing the stylus tip on a static dot (10 mm diameter) presented in the centre of the tablet's screen. After a second's delay the dot moved across the screen and participants were instructed to keep the tip of the stylus as close as possible to the dot's centre for the remainder of the trial. The motion was described by two oscillating sinusoidal waveforms in the axes of the screen. The frequencies and amplitudes of these waveforms were in a 2∶1 ratio, resulting in a repeating ‘figure-8’ spatial pattern (see Figure 1) with height  = 55 mm and width  = 110 mm. The trial required participants to track the moving dot for 84 seconds through a total of nine ‘figure-8’ revolutions comprising a ‘slow’ pace for the first three revolutions, transitioning to a ‘medium’ pace on the fourth revolution before transitioning to a ‘fast’ pace for the final three revolutions (i.e. a trio of revolutions at each successive speed). The frequencies specified for the waveforms in order to produce the three speeds and the resultant velocities of the dot are reported in Table 2. The second Tracking trial was identical to the first but the spatial path followed by the dot was provided in the background of the screen as a black 3 mm wide ‘guide’ line. This guide was expected to aid participants by providing additional information about the dot's path. See Figure 1a for illustrations of both trials. *Root mean square error* (*RMSE*), a measure of the spatio-temporal accuracy of participants' tracking, provided an index of performance on the tracking task. *RMSE* was calculated as the straight-line distance in millimetres between the centre of the moving target and the tip of the stylus for each sampled point during the time-series. For each Tracking trial (i.e. without and with guide-line) a mean value for *RMSE* with respect to each speed condition (i.e. a slow, medium and fast measure per trial) was calculated and statistically analysed.

**Table 2:** Frequency parameters for the three pattern speeds, plus resultant durations and velocities.

Pace	X-axis Frequency (Hz)	Y-axis Frequency (Hz)	Time per Figure-8 (sec)	Average Resultant Velocity (mm/s)	Minimum resultant Velocity (mm/s)	Maximum Resultant Velocity (mm/s)
*“Slow”*	0.125	0.0628	16	41.9	28.6	61.1
*“Medium”*	0.250	0.1250	8	83.8	57.2	122.2
*“Fast”*	0.500	0.2500	4	167.7	114.3	244.3

#### Aiming

The aiming subtest required 75 successive aiming movements to target-dots on the tablet's screen. Participants started by placing their stylus on the start position (a circle with the letter ‘S’ within it), triggering a target-dot (5 mm diameter) to appear at location 1 (see Figure 1b). Participants were instructed to respond as quickly and accurately as possible to this presentation by sliding their stylus across the screen to hit the dot. Arrival resulted in the dot disappearing and a new target-dot simultaneously appearing at location 2. Participants had to respond to this second target in the same manner as the first, in turn causing it to disappear and the next target-dot to appear at location 3. Participants repeated this pattern of response until the 75^th^ target, after which the finish position (a circle with the letter ‘F’ within it) appeared on screen (see Figure 1b). The overall sequence of 75 target-dot presentations encompassed two experimental conditions. The *Baseline* condition constituted the first 50 target-dot presentations. Within it target-dots cuing to each of the 5 numbered target locations were presented in order before location 1 was re-cued again the 5-step sequence repeated; ten times consecutively in the course of this condition (i.e. participants' resultant movements approximated drawing the star shape outlined in fourth panel of Figure 1b ten times in a row). Distance from one target location to the next was a constant 113 mm. The remaining 25 targets constituted the *Online Correction* condition, within which six ‘Jump’ events were pseudo-randomly programmed. On these movements, the target-dot instantaneously disappeared when the participant was within 40 mm of the intended target whilst another appeared simultaneously at the next-to-be-cued location in the established sequence. This required an online correction to their initial aimed movement. Participants were not explicitly told of the repeating pattern in the aiming movements or of the possibility jump-events would occur. Given the discrete nature of each aiming movement (i.e. only one dot on the screen at any one time), the rapid succession of responses to be made and the fact participants were instructed that upon arriving at a target-dot the next one could appear anywhere on the screen, it was expected that participants would treat each aiming as a discrete response (i.e. as opposed to a component of a greater, ultimately predictable, sequence). *Movement time* (*MT*) was calculated for each of the 75 discrete aiming movements and defined as the time between arrival at one target location and arrival at the next one (i.e. a composite measure of the time taken to prepare *and* then execute each aimed movement), in seconds. *MT* was calculated with respect to the *final* target position (i.e. after the dot had jumped) for Jump events. Fast *MTs* were indicative of an optimal task response). For statistical analysis, a median value for the *MT* of aiming movement made during *Baseline* experimental conditions was calculated. This was compared to two further median *MT* values derived from responses during the *Online Correction* condition. Within this condition a median *MT* value was calculated for the six aimed movements made in response to the ‘Jump’ events and a separate median was calculated for responses made to the interspersed normal stimuli presentations (termed the ‘Embedded-Baseline’).

#### Tracing

The Tracing subtest comprised six trials. In each trial the participant was required to place their stylus on the start position on an otherwise blank screen. After one second a tracing path (4 mm width) would appear, adjoining the start position to a finish position marked at the other end of the path (see Figure 1c). To complete the trial, participants had to move the stylus along the tracing path to the finish position; trying as best they could to stay within the path's guide-lines whilst doing this. The stylus produced an on-screen ‘ink trail’ (like a real pen), providing feedback to participants on their progress. Each trial presented one of two paths (A or B), which had identical geometry but were mirrored vertically (see Figure 1c). The paths were presented in alternate trials (path A on odd numbered trials and path B on even), meaning each was traced three times in total in the course of the subtest. In each trial, a black transparent box was presented on the screen next to the start position encompassing approximately one seventh of the length of the tracing path. At 5 second intervals, after the participant had begun tracing, this box shifted sequentially along the path, until after seven shifts (totalling 35 seconds) it arrived next to the finish position. Participants were explicitly instructed to try to remain within this box with their stylus whilst they were tracing along the path. The addition of this ‘pacing’ box was intended to standardise the speed (approximately), preventing variation in individual participants' prioritisation of ‘speed’ and ‘accuracy’ with respect to their performance from confounding results. *Path accuracy* (*PA*) for each trial was defined as the arithmetic mean (in mm) across all samples within each trial for the distance from the stylus to an idealised reference path (i.e. path A or B). Initial exploration of the data suggested that there was a degree of individual variation in the *movement time* (*MT*) within each of the Tracing trials (see Table 3). A composite metric was therefore created that adjusted participants' *PA* score to take account of their temporal accuracy. Thirty-six seconds was set as the optimum *MT* with each trial's *PA* score inflated by the percentage deviation from this time. This gave a new unitless measure combining estimates of spatial and temporal accuracy, called the *penalised path accuracy* (*pPA*) score. A median *pPA* value for participant's performance on the three Tracing trials presenting Path A and a separate one for the trials presenting Path B were calculated and analysed statistically (attempted statistical modelling of *pPA* as a repeated measure with an individual value for each of the six separate trials resulted in a model which failed to converge, hence separate summaries for A and B were instead analysed).

**Table 3:** Descriptive statistics for *movement time* (*MT*) during Tracing trials.

	movement time (in seconds)				
Trial Number	*Median*	*IQR*	*Range*	*n*±*5 sec* 1	*% n*±*5 sec* 1
1	38.3	36.4 to 41.4	26.8 to 93.9	119	28%
2	37.2	35.6 to 40.1	2.7 to 82.6	106	25%
3	37.4	35.4 to 39.8	20.3 to 72.3	104	25%
4	37.1	35.3 to 39.7	1.6 to 69.6	91	22%
5	37.1	35.3 to 39.4	2.8 to 70.1	102	24%
6	36.9	35.1 to 39.8	16.7 to 60.9	112	27%

1 Participants whose *MT* was either >41 seconds or <31 seconds (i.e. more than 5 seconds [i.e. 1 ‘pace box’ or more] adrift either side of the expected completion time).

## Results

All analyses were conducted in R (version 2.15.1, R Development Core Team, 2012). Primary outcomes for each subtest (*RMSE, MT* and *pPA*) were initially explored using graphs, skew and kurtosis values and Shapiro-Wilks tests of normality. Prior to statistical analysis reciprocal transformations were applied to all three outcome variables to normalise their distributions and resolve outliers. Performance on each of the transformed outcomes was then analysed separately using multi-level linear modelling (MLM) techniques (approximately equivalent to using mixed Generalised Linear Models); see Field [40] for a discussion of the advantages of MLM. All MLMs used a maximum likelihood method to estimate the model and specified Age Band (‘4 to 5’, ‘6 to 7’, ‘8 to 9’ and ‘10 to 11’) and Sex (Male or Female) as between-subject independent variables. Within the MLM model used to analyse *RMSE* (the primary outcome measure for the Tracking subtest) two additional repeated measures, both nested within participants, were also included to examine the influence of Trial Type (With- or Without-Guide) and Speed (Slow, Medium or Fast) respectively. Equivalently, for MLM analysis of *MT* (the primary outcome for Aiming subtest) a repeated measure of Response-type (i.e. Baseline, Embedded-Baseline or Jump Event) was included. Whilst modelling of *pPA*, the outcome measure for Tracing, specified a repeated measure of Path Type (i.e. Tracing Path A or B) instead.

A standardised protocol for conducting MLM analysis was followed [40]: First, a baseline model including no predictors except the intercept was generated. Next, a sequence of nested models was generated that added in, one at a time, the necessary pre-specified Main effects and associated interaction terms until a final full factorial model was reached. The effect of each Main Effect/Interaction term was then judged using likelihood-ratio tests which compared: (1) fit for the model in which a Main Effect/Interaction was included for the first time against (2) the fit for the immediately preceding model in the nested sequence. Thus, each likelihood-ratio test evaluated whether addition of a specific term (main effect or interaction) significantly increased the explanatory power of the model being built.

### Tracking (without and with a spatial guide)

**Figure 2:**
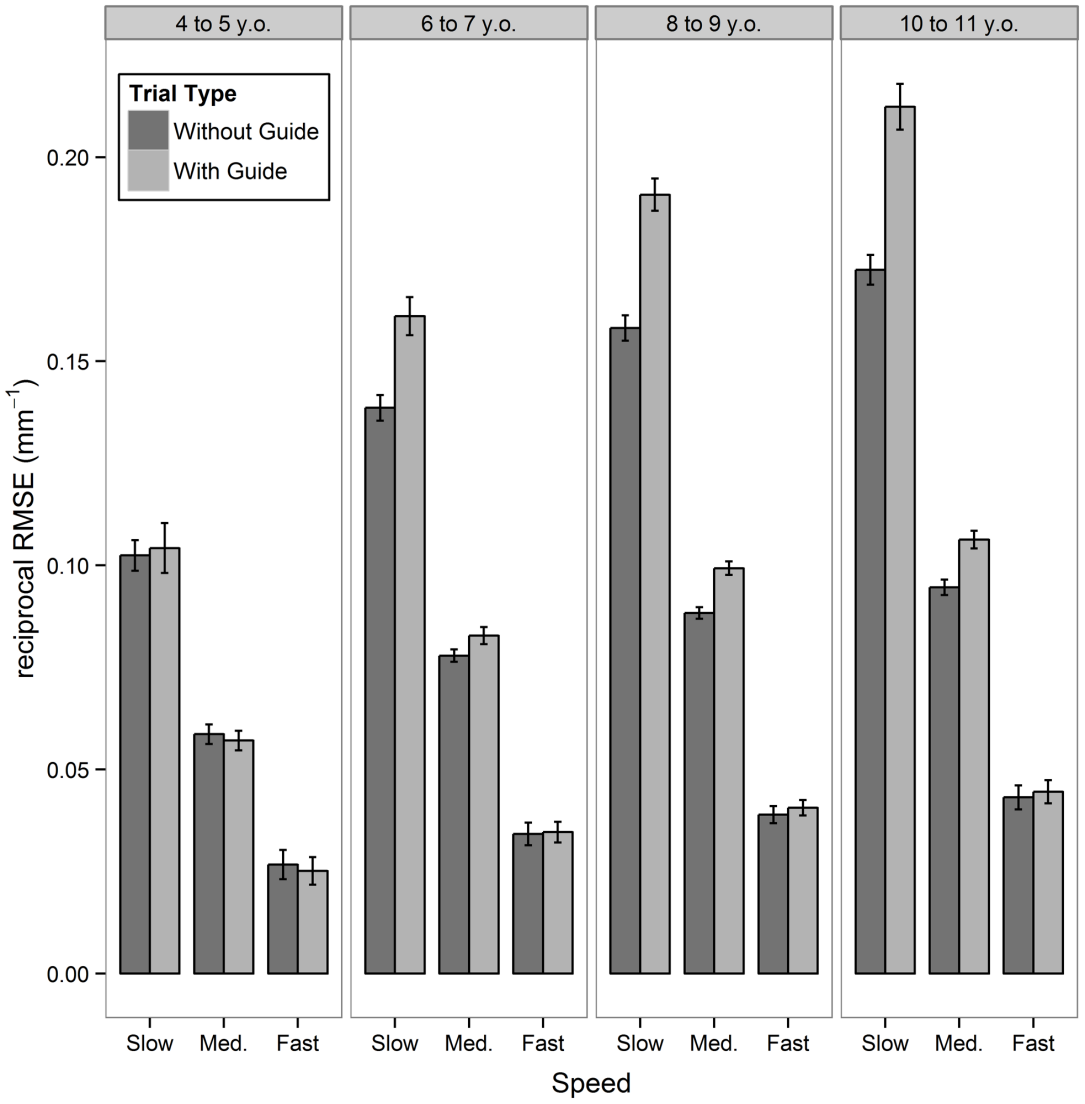
Bar-chart of reciprocal root mean square error (*RMSE*) by Age-Group, Trial-Type and Speed. Reciprocal *RMSE* (mm^−1^) is a measure of average spatial accuracy across time whilst manually tracking. Presentation of a guideline underneath the tracked target significantly improved performance on this outcome but this advantage was moderated by both age (larger benefit in older age groups) and speed (larger advantage at slower speeds), resulting in a statistically significant 3-way interaction between these factors (p<.001). There were no main effects or interactions involving Sex on this outcomes Note: Error bars represent 95% confidence intervals.

For two participants a recording error on this subtest meant their response had to be excluded from this portion of the analyses (leaving n = 420). MLM analysis of the reciprocal *RMSE* outcome found that the following 3-way interaction was significant: Age Band × Speed × Trial Type, (χ^2^(6) = 86.24; p<.001), depicted in Figure 2. All main effects and two-way interactions which involved only these three factors were also significant (p<.001). Meanwhile, the 4-way interaction that also included sex was non-significant (χ^2^(8) = 10.21; p = .251). No 3- or 2-way interactions involving Sex as a factor, or the main effect of Sex, were significant (all p>.499).

In relation to the significant 3-way interaction, Figure 2 suggests *RMSE* does not improve for the youngest Age Group when in the second trial the additional ‘guide-line’ is provided, irrespective of the speed of the dot. For older age groups their *RMSE*s do improve on the guide-line trial (higher scores  =  better after the reciprocal transform), with this benefit increasing with age but also diminishing as the target moves faster. This interpretation is supported by Table 4, which presents estimated effect sizes for performing with and without the guide-line for each age group at each speed. ‘Large’ sized benefits were found for tracking with the guide-line in three eldest age bands, when the target speed was Slow, with these benefits increasing successively with age. Similarly, ‘Small’ benefits, increasing to ‘Large’ with age, also emerged in these age bands when the target moved at the Medium speed. Effect sizes are interpreted using the threshold's suggested by Cohen (‘Small’ *d*>.20; ‘Moderate’ *d*>.50; ‘Large’ *d*>.80) [41].

**Table 4:** Mean and standard deviation for *reciprocal root mean square error* (*RMSE*) whilst Tracking without and with a guide-line, with effect size estimates for between-task differences.

		reciprocal RMSE (mm^−1^)				
		Without Guide-line		With Guide-line		
Age Band	Target Speed	mean	SD	mean	SD	Cohen's *d* 1
4 to 5 years	Slow	0.1005	0.0340	0.1023	0.0445	0.077
	Medium	0.0567	0.0152	0.0551	0.0243	0.110
	Fast	0.0247	0.0059	0.0232	0.0090	0.224
6 to 7 years	Slow	0.1373	0.0345	0.1598	0.0440	0.923
	Medium	0.0766	0.0176	0.0816	0.0232	0.348
	Fast	0.0329	0.0074	0.0334	0.0110	0.076
6 to 7 years	Slow	0.1571	0.0313	0.1898	0.0363	1.110
	Medium	0.0872	0.0150	0.0982	0.0180	0.836
	Fast	0.0379	0.0068	0.0396	0.0105	0.226
10 to 11 years	Slow	0.1705	0.0286	0.2104	0.0399	1.606
	Medium	0.0927	0.0155	0.1044	0.0200	0.941
	Fast	0.0413	0.0070	0.0426	0.0085	0.185

1 Effect size for the mean difference between reciprocal RMSE With and Without a Guide-line.

### Aiming

**Figure 3:**
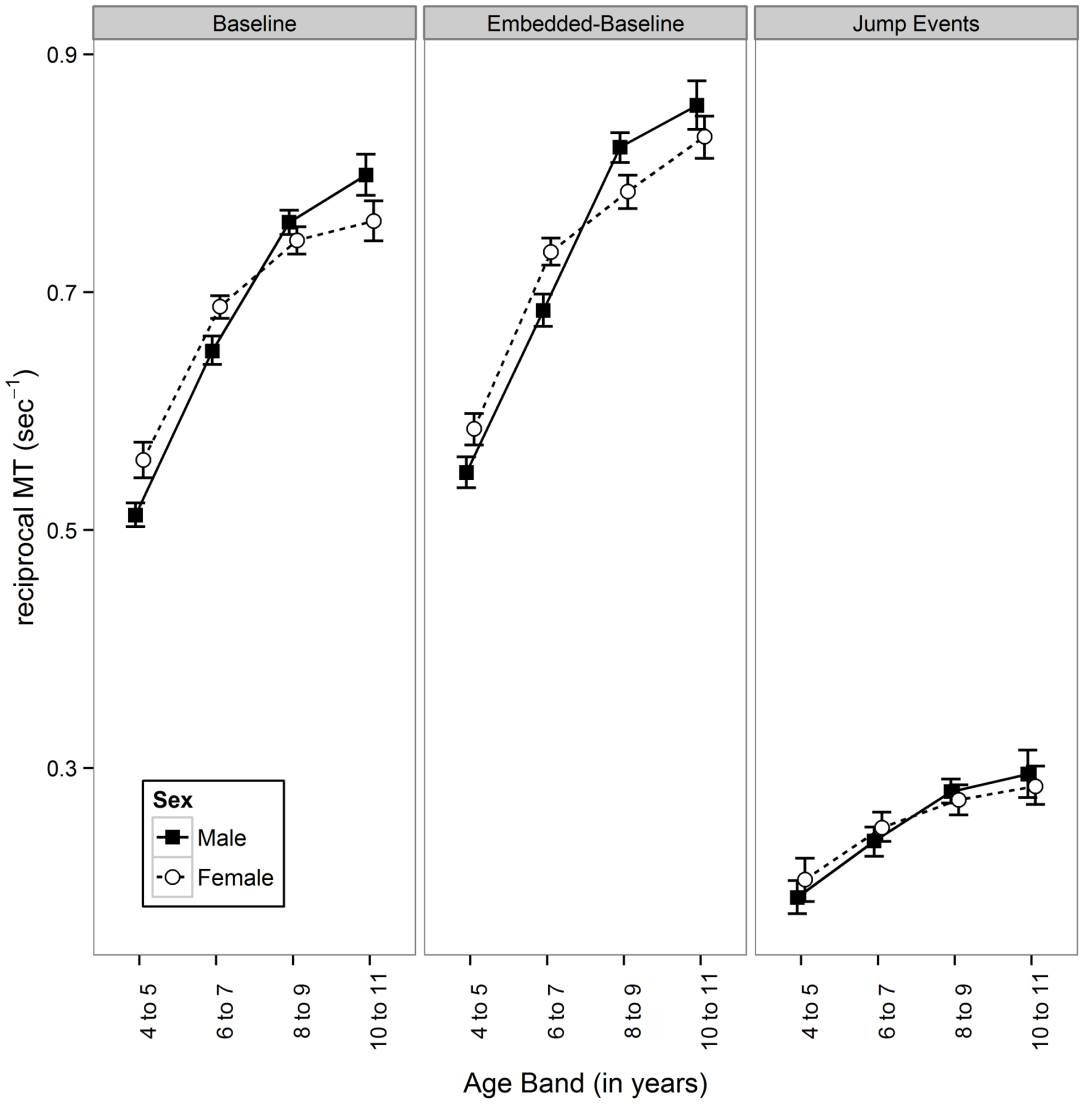
Line-graph of reciprocal movement time (*MT*) by Age-Group, Sex and Experimental Condition. Reciprocal *MT* (sec^−1^) is a measure of average time to move from one target to the next in a serial aiming task. In normal Baseline and Embedded-Baseline trials Female participants had a statistically significant advantage over males in the younger age-groups, with this crossing over in the older age-groups (i.e. no sex differences or a male advantage dependent on age-group and Condition). Meanwhile, no significant differences between sexes were observed, irrespective of age, for ‘Jump’ aiming movements that required additional online corrections. This was reflected in statistical analysis finding a significant 3-way interactions between Age-Group, Sex and Condition (p<.05). Note: Point estimates and associated 95% confidence Intervals for each sex group within an age-group have been artificially moved on the horizontal axis so that they display side-by-side, preventing overlaps obscuring interpretation.

One participant had only partial data recorded for the Jump condition and therefore their responses were excluded from this portion of the analyses. For the remainder of the sample (n = 421), MLMs of the reciprocal *MT* outcome revealed a significant 3-way interaction (depicted in Figure 3) for: Age Band × Sex × Response Type, (χ^2^(6) = 14.79; p = .022). Subordinate main effects for Age Band and Response Type and 2-way interactions for Sex × Age Band and Age Band × Response Type were also significant (all p<.008). All remaining main effects and interactions were non-significant (p>.857). Figure 3 shows evidence of sex differences arising in *MT* during Baseline and Embedded-Baseline trials but not during ‘jump’ events. In both these conditions a similar pattern is shown: a consistent female advantage in the youngest two age groups (4–5 and 6–7 year olds) which shows signs of reversing with age. In the older two age groups (8–9 and 10–11 year olds) there was either no significant sex difference within age-group or a significant male advantage. Table 5 investigates the magnitude of the sex-differences observed within this interaction, presenting descriptive statistics for male and female performance within each age-band on each condition. The corresponding effect-size for these mean differences indicate sex-differences constitute a ‘Small’ effect in terms of their magnitude (i.e. 0.2<*d*<0.5).

**Table 5:** Mean and standard deviation for *reciprocal movement time* (*MT*) whilst Aiming by Sex across Age Bands and Experimental Conditions, with effect size estimates for between-sex differences.

		reciprocal movement time (sec^−1^)				
		Males		Females		
Exp. Condition	Age Band	mean	SD	mean	SD	Cohen's *d* 1
Baseline	4 to 5 years	0.508	0.098	0.554	0.139	0.388
	6 to 7 years	0.648	0.094	0.685	0.108	0.366
	8 to 9 years	0.757	0.100	0.741	0.098	0.162
	10 to 11 years	0.795	0.124	0.757	0.124	0.306
Embed. Base.	4 to 5 years	0.543	0.115	0.580	0.129	0.303
	6 to 7 years	0.682	0.105	0.732	0.120	0.444
	8 to 9 years	0.820	0.120	0.783	0.118	0.311
	10 to 11 years	0.854	0.139	0.828	0.124	0.198
Jump Events	4 to 5 years	0.186	0.037	0.202	0.045	0.390
	6 to 7 years	0.236	0.034	0.247	0.043	0.286
	8 to 9 years	0.279	0.035	0.271	0.034	0.232
	10 to 11 years	0.292	0.039	0.282	0.035	0.270

1 Effect size for the mean difference between sex for reciprocal MT.

### Tracing

**Figure 4:**
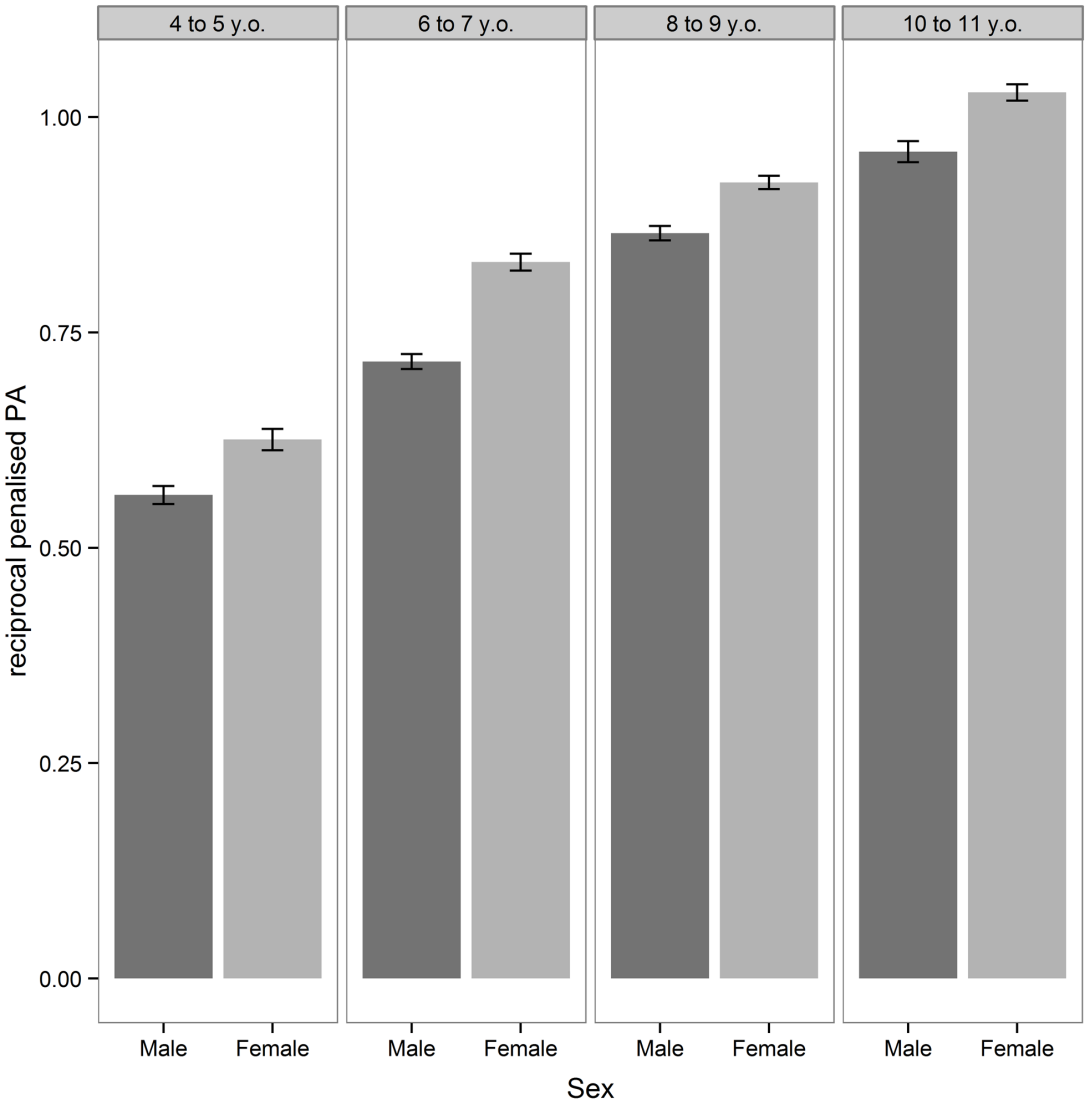
Bar-chart of reciprocal penalised path accuracy (*pPA*) by Age-Group and Sex. Reciprocal *pPA* is a unitless measure of spatial accuracy whilst tracing, adjusted to standardise for individual variation in speed. Statistically significant differences between Age-Groups and Sex were found on this outcome (both p<.001), with no significant interaction between them. Performance improved with increasing age and was consistently better (higher) in Females. Note: Error bars represent 95% confidence intervals.

Multilevel linear modelling found significant main effects of both Age Band (χ^2^(3) = 259.57; p<.001) and Sex (χ^2^(1) = 15.25; p<.001) upon reciprocal *pPA* but no additional significant main effect for Path type (A or B) (χ^2^(1) = 1.95; p = .165) or any significant 2- or 3-way interactions (all p>.409). Inferring from descriptive statistics, the main effect of sex indicated Girls' mean reciprocal *pPA* score was significantly higher (better) than boys (Girls: mean [SD]  = 0.854 [0.220]; Boys: mean [SD]  = 0.780 [0.226], *d* = 0.332). Post-hoc tests also showed that from one Age band to the next 6-to-7 year olds out-performed 4-to-5 year olds (Mean Diff. [95% CI]  = 0.154 [0.074 to 0.235]; p<.001, *d* = 1.008), 8-to-9 year olds were better than 6-to-7 year olds (Mean Diff. [95% CI]  = 0.153 [0.085 to 0.221]; p<.001, *d* = 0.650) and 10-to-11 year olds outdid the 8-to-9 year olds (Mean Diff. [95% CI]  = 0.100 [0.021 to 0.179]; p = .007, *d* = 0.569). Effect sizes suggested ‘Moderate’ to ‘Large’ sized improvement with Age but only a ‘Small’ sized effect for Sex. See Figure 4 for an illustration of these effects.

## Discussion

Our study explored pre-pubescent (4–11 years old) children's manual control, using a set of tasks that reflected the basic eye-hand coordination challenges commonly encountered when engaging in such behaviour. We restricted our attention to the control of a stylus held in the hand and explored three separate tasks that had different control demands: aiming movements, tracking and tracing. Our findings provide the first detailed evaluation of the degree to which sex differences influence the development of manual control within this age range. Girls exhibited better performance on the aiming and the tracing tasks but the higher performance observed in the aiming task was restricted to the youngest age groups. These female advantages are broadly consistent with previous studies, which used non-computerised assessments and reported girls outperforming boys on tests of manual dexterity (7 to 12 years old) [20], [28]. The results also agree with research that has found girls outperform boys on pencil-and-paper based standardised assessments of handwriting ability [42], [43], handwriting being a skill that is partly contingent on an individual's underlying manual dexterity [10]. Such findings all corroborate the widely held perception that girls are predisposed to perform better than boys on precision manual control tasks, such as handwriting - a belief supported by research showing participants rate female handwriting samples as neater even when blinded to the sex of the writer [44]). Pre-school development of fine-motor skills have been suggested to progress at a faster rate in females [45], [46], thus it is plausible that during pre-pubescence males continue to lag behind their female counterparts, requiring more time to mature and acquire certain manual control abilities.

The emergence of a male advantage in the older age groups for aiming suggests that the magnitude and direction of sex-differences has the potential to change over the course of development. The interaction observed is consistent with other studies that have shown a male advantage from adolescence onwards for producing fast, simple manual responses (e.g. finger-tapping and simple reaction times) [2], [47]. It also parallels male advantages in gross-motor object control skill beginning in adolescence [11], [19]–[23]. Studies in adults also suggest males are better at making precise aiming movements (on both gross and fine tasks) and have an advantage in motor tasks that require arm as opposed to hand movement [48], [49]. Given the nature of the aiming task we used, it is reasonable to assume it may have necessitated more ballistic-type movements than tracking or tracing (i.e. it was the only task in the battery intended to induce rapid movement from one discrete point of the screen to the next) and in turn it is plausible this may have required more upper arm involvement in controlling these particular movements.

The causes of these emerging male-advantages cannot be resolved from our study. Parsimonious explanations for why male advantages might emerge include: increasing anatomical differences in males and females (e.g. greater musculature) [24], differences in the neural pathways the sexes use to execute motor control [50], which leads to disparities in cognitive processing speeds for certain sensorimotor tasks [47] or experience/practice induced effects arising from males involving themselves more in activities that necessitate precise aiming (e.g. sports, computer game use) [22], [51]. Equally, sex-differences in motor sequence learning ability might explain the results. There is some evidence of males outperforming females on motoric tasks that require learning of a novel finger tapping sequence, with this advantage increasing with age (9 to 17 years old) [26]. The aiming task had a repeating pattern within its movements (i.e. five points of a star repeated 15 times), which participants were not made explicitly aware of. Nevertheless, more attentive participants may have become aware of this implicit pattern in the course of performing the task and been able to use it to their advantage (i.e. could use the pattern to predict where the next target location would be). Given the suggested male advantage for learning manual sequences it is plausible that older males, who became aware of the learning element embedded in this task, may have selectively benefitted to a greater degree.

In contrast to the aiming and tracing task, there were no sex differences in the tracking task. It is always difficult to interpret a null finding but the fact that differences emerged on the other two tasks suggests that any diversity between the sexes on the tracking task must be very small if it exists at all. Tracking tasks are known to be sensitive indicators of certain neurological deficits because they rely on corticocerebellar and visuomotor control systems to generate accurate predictions of an external target's motion [52]–[54]. Thus, a limiting constraint on tracking performance is an individual's ability to predict target motion, meaning that sex differences in manual control might be masked because of an upper limit on motion prediction. It has been reported previously that the normal right-left hand performance asymmetry is not found on manual tracking tasks for this reason [55]. If we consider all of these findings together, one might speculate that our results suggest girls, pre-adolescence, have a marginal advantage over boys for handling a stylus and exerting precise force-control upon it (as indexed by the tracing task) but that competing tasks demands may swamp this advantage, leading to superior performance disappearing when manual tasks also contain other constraints (e.g. a reliance on predictive neural circuits or motor sequence learning).

Our findings sound a note of caution for past and future studies that explore sex differences using complex ‘fine motor tasks’ (e.g. handwriting) in part because such tasks become more prone to the effects of experience but also because such tasks contain different control elements [10] that might exert different effects beyond the researcher's control. Manual tasks are real observable behaviours (e.g. drawing, writing) that are likely to be subject to extensive practice from an early age and dependent on a wide range of cognitive functions in addition to manual control alone [10]. Nonetheless, there are certain discrete abilities that are likely to be frequently required when performing manual behaviours (e.g. predicting a target's movement, exerting precise force control on a handheld tool, using accurate feedforward mechanisms for fast implementation of online corrections), which we have endeavoured to explore via our specific set of novel tasks. Therefore, our battery is likely to be representative of sex-differences in *most* manual behaviours involving a handheld stylus but it would be overly reductive to claim it should be taken as a proxy for *all* manual behaviours. Specifically, our battery is likely to be most relevant to understanding the underlying functions that contribute to an individual's proficiency in educationally important activities requiring manual stylus use, such as handwriting, drawing and touch-screen computer use.

The fact that we have found sex differences in manual control raises the issue of whether the disparities warrant different educational approaches to handwriting tuition. This requires consideration of the sizes of the sex-differences observed: the absolute differences and associated standardised effect sizes we report are generally ‘small’, when judged against conventional thresholds [41]. A consideration of their size relative to other measurable influences on manual control suggests they may be more noteworthy: the sex effect size on tracing equates to between 33 and 58% of the size of the year-on-year improvements with age in tracing performance. But this was the single most consistent sex effect and it still only implies that at worst boys might perform at a level a few months behind that of their female peers (i.e. a manageable discrepancy within the classroom). The tasks we used were novel in nature and not culturally dependent. This gives us some confidence that our study has elucidated underlying control differences between the sexes. Nevertheless, it is impossible to be certain that our findings do not reflect culturally imposed differences in developmental history. Also, the cross-sectional design we employed does not allow us to discern whether there are sex differences in the rate of learning of different novel manual skills (an important future research question). Setting to one side questions of how manual control differences may arise, our current results suggest that, given their magnitude, it is hard to argue that boys should receive different educational opportunities than girls. In the context of our earlier introduction, the findings favour a ‘gender-similarities’ hypothesis [6]. They demonstrate that sex-differences in the motor, as in the cognitive domain, are highly task-specific and small in magnitude. This cautions against over-interpreting such disparities as reductive explanations for why differences in educational performance may arise between the sexes in the general population [7], [9].

Finally, we should emphasise that the present study has focussed exclusively on population differences (we deliberately applied transformations to ensure the normal distribution of our outcome measures, accounted for outliers and used powerful statistical techniques that were robust to any violations of the homogeneity of variance assumption). There are good reasons to suppose that at an individual level there will be more boys than girls who have specific problems with eye-hand coordination [8]. Developmental Coordination Disorder (DCD) is more common in boys than girls, with estimates of the exact ratio ranging between 2∶1 [17] and 7∶1 [18]. However, our findings do not support the interpretation of DCD as simply a characterisation of the motor skills of those children at one end of a continuum within the population [56], [57], which is consistent with a large number of studies that indicate pathological causes for DCD [58], [59]. Children with DCD undoubtedly need additional educational support [60] but this should be based on identifying a child with a special need regardless of their sex. Individual differences in manual control are much greater than the relatively small differences we have identified between boys and girls, as predicted by the gender similarities hypothesis [61].
