# Correction: Manual Control Age and Sex Differences in 4 to 11 Year Old Children

**DOI:** 10.1371/journal.pone.0097981

**Published:** 2014-05-15

**Authors:** The PLOS ONE Staff

There is an error in Table 4. The Age Band column incorrectly lists the age 6 - 7 years twice. Please find a corrected version of Table 4 below.

**Table 4:** Mean and standard deviation for reciprocal root mean square error (RMSE) whilst Tracking without and with a guide-line, with effect size estimates for between-task differences.

		reciprocal RMSE (mm^-1^)				
		Without Guide-line		With Guide-line		
Age Band	Target Speed	mean	SD	mean	SD	Cohen’s *d* 1
4 to 5 years	Slow	0.1005	0.0340	0.1023	0.0445	0.077
	Medium	0.0567	0.0152	0.0551	0.0243	0.110
	Fast	0.0247	0.0059	0.0232	0.0090	0.224
6 to 7 years	Slow	0.1373	0.0345	0.1598	0.0440	0.923
	Medium	0.0766	0.0176	0.0816	0.0232	0.348
	Fast	0.0329	0.0074	0.0334	0.0110	0.076
8 to 9 years	Slow	0.1571	0.0313	0.1898	0.0363	1.110
	Medium	0.0872	0.0150	0.0982	0.0180	0.836
	Fast	0.0379	0.0068	0.0396	0.0105	0.226
10 to 11 years	Slow	0.1705	0.0286	0.2104	0.0399	1.606
	Medium	0.0927	0.0155	0.1044	0.0200	0.941
	Fast	0.0413	0.0070	0.0426	0.0085	0.185

1 Effect size for the mean difference between reciprocal RMSE With and Without a Guide-line.
